# Evaluation of Mesoporous TiO_2_ Layers as Glucose Optical Sensors

**DOI:** 10.3390/s22145398

**Published:** 2022-07-20

**Authors:** David Ortiz de Zárate, Sara Serna, Salvador Ponce-Alcántara, Jaime García-Rupérez

**Affiliations:** Nanophotonics Technology Center, Universitat Politècnica de València, Camí de Vera s/n, 46022 Valencia, Spain; sasermo@etsii.upv.es (S.S.); salponce@ntc.upv.es (S.P.-A.)

**Keywords:** optical sensor, mesoporous layer, bottom-up fabrication, glucose sensing, sol-gel, TiO_2_, Fabry–Pérot interferometer

## Abstract

Porous materials are currently the basis of many optical sensors because of their ability to provide a higher interaction between the light and the analyte, directly within the optical structure. In this study, mesoporous TiO_2_ layers were fabricated using a bottom-up synthesis approach in order to develop optical sensing structures. In comparison with more typical top-down fabrication strategies where the bulk constitutive material is etched in order to obtain the required porous medium, the use of a bottom-up fabrication approach potentially allows increasing the interconnectivity of the pore network, hence improving the surface and depth homogeneity of the fabricated layer and reducing production costs by synthesizing the layers on a larger scale. The sensing performance of the fabricated mesoporous TiO_2_ layers was assessed by means of the measurement of several glucose dilutions in water, estimating a limit of detection even below 0.15 mg/mL (15 mg/dL). All of these advantages make this platform a very promising candidate for the development of low-cost and high-performance optical sensors.

## 1. Introduction

Sensors offer humans an extended ability to interact with their environment, enabling the development of new devices that can make our lives better, easier and safer. The detection and quantification of different types of substances/analytes, such as proteins, antibodies, DNA/RNA, pathogens, antibiotics, allergens or contaminants, is of utmost importance in a wide range of application fields such as environmental monitoring [1,2], food safety [3,4], healthcare [5,6], defense [7,8] or drug development [9,10], among many others.

Different transduction mechanisms are typically used in the development of sensing devices (electrical, mechanical, chemical, etc.), but optical sensors stand out from among them because of their remarkable advantages: high sensitivity, high level of miniaturization, rapid results, very low volumes of required sample and reagents needed, label-free detection, resistance to hazardous and harsh environments, and immunity to electromagnetic interference [11]. The operation principle of optical sensors is typically based on measuring changes produced in the optical signal (amplitude, phase, polarization, etc.) when a variation in the refractive index is produced as a result of the presence of target substances/analytes in the surroundings of the optical structure. In many cases, only a small portion of the optical signal that is not confined within the optical structure is used for sensing, the so-called evanescent field, which can limit the performance of the sensing structure. As a consequence, there is a growing interest in the development of new optical structures that are capable of maximizing the interaction between light and the target substance/analyte. Following these guidelines, the unique physicochemical properties of nanostructured materials in comparison with their bulk counterparts (high surface-to-volume ratio, small size or tunable refractive index, among many others), make them an interesting option for developing novel sensor configurations that exhibit enhanced performance [12,13].

Among the different nanostructured materials, interest in using porous materials for the development of optical sensing structures has significantly increased during the last years. Firstly, porous materials allow the target substance to directly penetrate the sensing structure, thus increasing the amount of analyte interacting with the optical signal (not only with its external surface) and leading to a further increase in its sensitivity. Thus, the higher the porosity, the higher the sensitivity will be, but we will have to take the structural stability into account when using very high porosities. Secondly, the 3D porous architecture of these materials allows increasing the specific surface available for its biofunctionalization with biorecognition elements, thus maximizing the number of specific sensing interactions that can be detected. For a certain porosity value, the internal surface-to-volume ratio will inversely depend on the pore size. Hence, smaller pores will be preferred for biosensing purposes, but we will have to take into account that a minimum size will be required for the analytes/molecules to be able to enter the porous structure.

Most research that focuses on the development of optical sensors is based on porous materials that make use of top-down fabrication approaches for the creation of these porous substrates, as is the case for porous silicon (pSi) [14,15,16,17] and anodic aluminum oxide (AAO) [18,19,20]. These types of porous sensors exhibit several interesting properties, such as high sensitivity, good stability and chemical compatibility with different biofunctionalization routes, which make them suitable for many applications. The production of these porous substrates implies the anodization of the constitutive bulk material (e.g., silicon, aluminum) by the application of an electric current in an acid solution, in order to create the porous structure [21,22,23,24]. However, the use of these top-down fabrication strategies might lead to certain limitations [25]. For example, pores created by anodization are mainly etched in the vertical direction, and they are usually poorly interconnected (or even completely isolated). This leads to closed-end pores that can be difficult to fill with the liquid sample to be analyzed, thus reducing the diffusion of the target substance/analytes into the porous layer, and limiting its practical sensing performance. Also, the porous layer might present inhomogeneities in its structural properties (e.g., pore size, porosity, thickness) both in depth, due to the change in the etching conditions when going deeper into the layer, as well as along its surface, which results from the difficulty in obtaining a homogenous current density distribution across the entire substrate surface. This inhomogeneity problem will be especially relevant when pursuing the creation of porous layers over large substrates in order to increase the throughput and reduce the production cost, hence limiting their mass production potential and therefore their practical application. In order to overcome these limitations, other approaches based on the bottom-up synthesis of the porous substrates have been proposed. For example, the possibility of creating optical sensors based on a network of polymeric nanofibers fabricated by electrospinning, a technique suitable for mass production, has been recently demonstrated [26]. However, the low refractive index of the polymeric materials required for the electrospinning process makes the spectral features used to perform the sensing broader, and with a smaller amplitude than for their pSi and AAO counterparts; this increases the measured noise and reduces the achievable limit of detection.

Within this context, we have developed a bottom-up synthesis process for the creation of TiO_2_ mesoporous layers with a high degree of interconnectivity and homogeneity for the development of optical sensors based on Fabry–Pérot interferometry [27]. Selecting TiO_2_ as the inorganic material of the porous framework ensures a high refractive index for the porous scaffold (*n*_TiO2_ ≈ 2.56), hence enabling the porous layer to count on a sufficiently high effective index to provide an adequate optical response. Besides, TiO_2_ offers additional assets that are of interest in the field of biosensing, such as high mechanical and chemical stability, biocompatibility and self-cleaning ability through UV-light exposure, among others [28,29]. The synthesis process has been based on both the sol-gel route [30,31] and the evaporation induced self-assembly (EISA) [32] methods, since they can provide better control and homogeneity over the size and arrangement of the mesopores, even over large areas, thus enabling the potential mass production of the substrates at a low cost. 

The application of the fabricated mesoporous TiO_2_ layers as optical sensing structures has been evaluated by means of glucose sensing experiments, which potentially have an interest in different areas. In the biomedical field, sensors are tremendously important for monitoring glucose levels in patients with diabetes [33,34], a metabolic disorder that is affecting an increasing number of people around the world. In relation to the food industry, the application of sensors allows the determination of the glucose content of certain products, such as soft drinks, whose intake is one of the leading sources of added sugars in some countries’ diets, and increases the risk of obesity and diabetes [35]. In this paper, the potential use of the obtained mesoporous TiO_2_ layers as low-cost optical sensing structures was assessed using the direct detection of synthetic samples of glucose in water having concentrations ranging from 2.7 mg/mL (270 mg/dL) to 100 mg/mL (10,000 mg/dL); this covers the range typically found in beverages [36]. From the experimental results obtained, a limit of detection (LOD) even below 0.15 mg/mL (15 mg/dL) was estimated, which would also make the sensor potentially useful for monitoring glucose levels in less concentrated samples, such as those from blood or urine (e.g., typical concentrations between 50–500 mg/dL in blood samples [37]).

## 2. Materials and Methods

The synthesis of mesoporous TiO_2_ layers implied the use of several chemical reagents, all of which were used as received, without further purification. Acetone and isopropanol were supplied by Scharlab (Barcelona, Spain); titanium tetraisopropoxide, TTIP, was provided by TCI (Tokyo, Japan); Pluronic P123 was gently supplied by Carbosynth Limited (Berkshire, UK); and hydrochloric acid and 1-butanol were provided by Sigma-Aldrich (Burlington, VT, USA). Siegert Wafer supplied the silicon substrates over which the mesoporous TiO_2_ layers were created, and these were sliced into pieces of about 15 × 15 mm^2^ by means of an automatic dicing saw Disco DAD3350 (Disco Co., Tokyo, Japan).

### 2.1. Synthesis of the TiO_2_ Sol

A stable homogeneous sol was prepared by following the method previously reported, with some modifications [27]. The sol synthesis was conducted by the initial hydrolyzation of TTIP in the acidic mixture of 1-butanol, hydrochloric acid and water, counting also with the P123 (EO_20_PO_70_EO_20_) block copolymer. P123 templating agent was chosen because it provides bigger pores than other templating agents, which is a key parameter for biosensing (to have pores being big enough to avoid their blockage when biofunctionalizing them, and to allow for the penetration of target molecules/analytes into them, as previously indicated). The subsequent condensation step will only take place when the evaporation induced by the spin coating process reduces the amount of solvent (including HCl) present in the sol, making it less acidic and hence more reactive.

A molar ratio of 1 TTIP : 2 HCl : 0.013 P123 : 9 1-butanol was employed for the preparation of the TiO_2_ sol. In a conventional synthesis, concentrated hydrochloric acid (1.34 mL) was carefully mixed with TTIP (2.56 mL) under magnetic stirring at room temperature, in order to prevent the immediate precipitation of a white TiO_2_ powder. Meanwhile, a second solution containing the P123 surfactant (0.65 g) and 1-butanol (6 g) was prepared at room temperature under vigorous stirring. In this mixture, P123 acts as the template for the creation of the porous network, while 1-butanol is responsible for enhancing the phase separation between the template and the inorganic framework as well as promoting the expansion of P123 micelles to enlarge the pore size. After the complete dissolution of the surfactant, the second solution was mixed with the first one, and it was magnetically stirred for 30 min at room temperature, promoting sol aging.

### 2.2. Fabrication of the Mesoporous TiO_2_ Layers

Before the deposition of any mesoporous TiO_2_ layer, the silicon substrates were cleaned by three ensuing rinses with acetone, isopropanol and deionized water, followed by a drying step with nitrogen gas flow. After this, an EVG101 spin coater (EVG, St. Florian am Inn, Austria) placed in a class 100 cleanroom was employed to homogeneously deposit the aged sol over the substrates, using a spinning speed of 3000 rpm for 15 s (temperature 20 °C/relative humidity 45%). An overnight aging process at room temperature under a relative humidity of 60% was then applied to the obtained layers, enabling their mesostructuration. The mesoporous TiO_2_ layers were then fabricated by means of a thermal treatment, which enhanced the mechanical and thermal resistance of the layers and also removed the organic surfactant, providing highly uniform mesoporous layers. This calcination process, carried out in an oven (Gallur, Manises, Spain) under air atmosphere, consisted of increasing the temperature up to 300 °C at a rate of 1 °C/min, in order to promote higher crosslinking of titania, and then holding the temperature for 1 h to ensure the complete thermal degradation of the template.

Finally, note that multilayer mesoporous TiO_2_ substrates were also prepared by subsequent processes of spin-coating deposition and aging before the final thermal treatment.

### 2.3. Characterization of the Mesoporous TiO_2_ Layers

A Veeco Dektak150 profilometer (Veeco, Plainview, NY, USA) was used to measure the thickness of the porous layers. A Zeiss GeminiSEM 500 (Zeiss, Oberkochen, Germany) high resolution field emission scanning electron microscope (HRFESEM) operating at low voltage (0.02–2 kV) was used to visually inspect the porous layers in order to determine characteristics such as their porous nature, pore arrangement and homogeneity, among others. A homemade platform working in the VIS-NIR range (450–900 nm) was used to measure the optical reflectance response of the porous layers. This platform comprises a halogen lamp used to illuminate the sample, and a spectrometer Ocean Optics Flame T (Ocean Optics, Orlando, FL, USA) used to obtain the reflection spectrum. Fabricated porous layers were modelled using the Transfer Matrix Method [38] and the Bruggeman effective medium theory [39], in order to estimate their effective refractive index and porosity.

### 2.4. Glucose Optical Sensing Experiments Using Mesoporous TiO_2_ Layers

Once fabricated and characterized, the mesoporous TiO_2_ layers were evaluated as optical sensing structures. For a given thickness and refractive index of the porous layer, it will present a certain Fabry–Pérot interferometric response that is determined by the wavelength-dependent constructive and destructive interference of the light reflected at the interfaces of that layer. The position of these spectral maxima and minima will shift when a change in the refractive index of the porous layer is produced by the presence of the target substance inside it, hence enabling the sensing.

In order to deliver the different glucose solutions to the sensing layers in a controlled way, a homemade microfluidic cell was placed on top of the mesoporous TiO_2_ samples, and a syringe pump working in withdraw mode at a rate of 20 μL/min was used to flow the liquid. Optical reflectance spectra were continuously acquired in the VIS-NIR range with the aim of determining the position of the interferometric lobes and tracking their shift when the glucose concentration was changed. Acquired spectra were processed using Matlab (MathWorks, Natick, MA, USA). The selected interferometric lobe was fitted to a sine function in order to determine its position with a higher accuracy. The noise level of the sensing signal was determined by calculating the standard deviation (σ) of the noise signal, which can be obtained by subtracting the sensing signal from its average (calculated using a moving average filter with a span of 10 points).

## 3. Results

**Figure 1:**
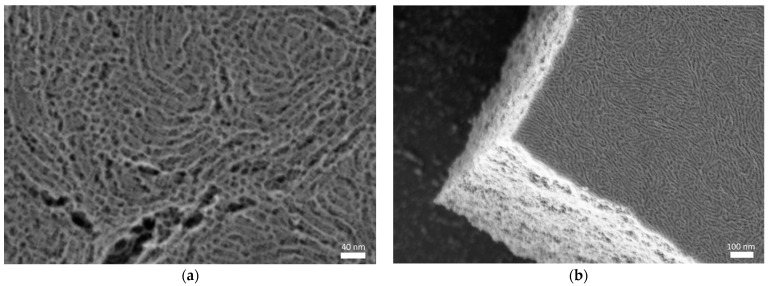
HRFESEM images of fabricated P123-templated mesoporous TiO_2_ layers. (**a**) Top view of a monolayer. (**b**) Cross section of a multilayer where the deposition and aging processes are repeated three times before the final thermal treatment.

**Figure 2:**
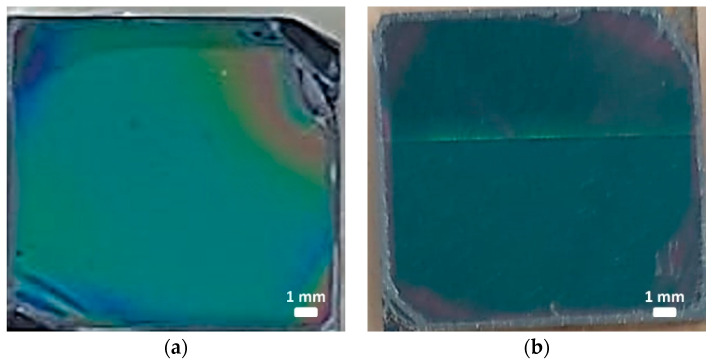
Optical images of fabricated P123-templated mesoporous TiO_2_ layers. (**a**) Monolayer. (**b**) Tri-layer. The size of each sample shown in these pictures is 15 × 15 mm^2^.

Figure 1a shows a HRFESEM image of the fabricated P123-templated mesoporous TiO_2_ layer after the aging and thermal treatment processes. The image displays a disordered arrangement of interconnected pores with an average diameter of around 15 nm, thus confirming stabilization of the three-dimensional porous structure after the thermal treatment. On the other hand, Figure 1b shows a HRFESEM image of a P123-templated mesoporous TiO_2_ multilayer where the deposition and aging processes were repeated three times before the final thermal treatment, confirming the possibility of increasing the thickness of the final mesoporous TiO_2_ layer by iteratively repeating the deposition and aging processes. The thickness of both types of samples (i.e., monolayer and tri-layer) was characterized using the profilometer, obtaining values of 210 and 651 nm, respectively. Regarding the homogeneity of the layers, Figure 2 shows that they display a uniform color over most of their surfaces, which indicates a high degree of homogeneity. Only a few inhomogeneities are observed at the edges of the sample, which are typical of the spin-coating process used for the deposition of the layers. The relative size of this inhomogeneous region can be reduced by applying the synthesis process over larger substrates in order to increase the yield of the fabrication process.

**Figure 3:**
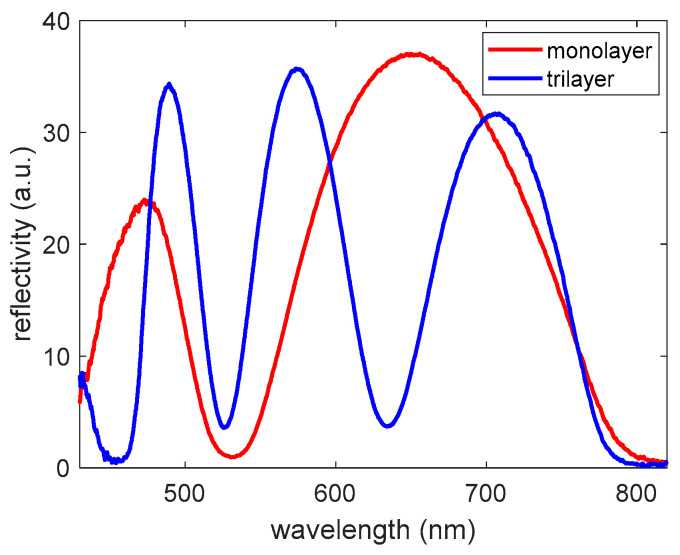
Comparison of the optical reflectance spectra in air for the mesoporous TiO_2_ monolayer (thickness = 210 nm) and tri-layer (thickness = 651 nm).

The fabricated mesoporous layers were optically characterized by measuring their reflection spectra with the optical setup previously described. As it is depicted in Figure 3, both samples clearly presented Fabry–Pérot interferometric responses, which were subsequently used to perform the sensing experiments by tracking the position of the layers’ lobes while infilling different substances within the pore network. As expected, the increase in the thickness that was achieved by creating a multilayer (tri-layer, in this case) promoted an increase in the number of interference fringes appearing in the spectrum, which become narrower and easier to track in a more accurate way when performing the sensing. Based on this, the fabricated tri-layer was used for the glucose sensing experiments. On the other hand, the high amplitude of the interference fringes is a consequence of the high effective refractive index of the mesoporous layer, which is what determined the use of TiO_2_ as their constitutive material.

**Figure 4:**
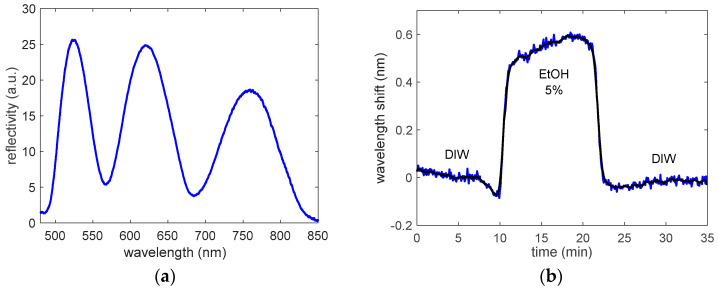
(**a**) Initial spectrum of the P123-templated mesoporous TiO_2_ tri-layer in DIW. (**b**) Spectral shift of the interference maximum of the reflection spectrum located at 620 nm when a concentration of 5% EtOH in DIW was flowed through the porous sensor. The raw sensing curve is depicted in blue color and the averaged sensing curve is depicted in black color.

In order to perform the sensing experiments with the mesoporous TiO_2_ tri-layer, the sample was assembled with the microfluidic flow cell previously described. In this way, the evolution of the optical reflectance spectrum of the 3D porous sensing layer could be continuously monitored when changing the substance that flowed through it. In the first stage, the bulk sensitivity and the LOD of the fabricated porous sensor were characterized by sequentially flowing deionized water (DIW) and a concentration of 5% in volume of ethanol (EtOH) in DIW. The initial spectrum of the porous sensor in DIW and the evolution of the position of the interference maximum located at 620 nm are shown in Figure 4. It can be observed that the target substances can perfectly enter into the porous sample, since an immediate spectral shift was produced as soon as the liquid reached the sensor. A wavelength shift of 623 pm was measured for the refractive index change induced by the 5% EtOH solution (Δ*n* = 2.4 × 10^−3^), thus leading to a bulk refractive index sensitivity (Δ*λ*/Δ*n*) of 260 nm/RIU (≡ Refractive Index Units). The high sensitivity and the fast optical response are in agreement with a mesoporous TiO_2_ layer displaying a high porosity, sufficiently large pore sizes and a remarkable pore interconnectivity. Despite being high, the obtained bulk sensitivity is somewhat smaller than that reported for other more typical porous substrates when working in the VIS range, as for the cases of porous silicon (e.g., ~350 nm/RIU [17]) or anodic aluminum oxide (e.g., ~450 nm/RIU [20]). However, note that these works use relatively complex structures based on concepts such as multilayer Bragg stacks, microcavities or graded refractive index profiles, while our structure is simply a triple layer of the same porous material. Therefore, a proper design of the porous configuration fabricated using the proposed bottom-up synthesis approach would allow us to increase the sensitivity, obtaining values comparable or even higher than those reported for more typical porous substrates.

The noise level of the measurement was determined for the DIW baseline obtained after the flow of EtOH 5%, obtaining a value of σ = 9.8 pm, thus leading to an estimated LOD of 1.13 × 10^−4^ RIU (calculated as 3σ/sensitivity). Note that the interference maximum located at 620 nm was selected to perform the spectral tracking because it is the interferometric fringe that provides the best balance between sensitivity and noise, and thus, provides the lowest LOD value. Spectral lobes located at longer wavelengths provide a higher sensitivity, but the noise of their sensing signals is also higher due to their greater broadness; for example, the interference maximum located at 760 nm provides a sensitivity of 303 nm/RIU with a noise of σ = 26.7 pm, leading to an estimated LOD of 2.65 × 10^−4^ RIU. Regarding spectral lobes located at shorter wavelengths, besides having a lower sensitivity, their sensing signals have a slightly higher noise that is determined by the lower intensity of the light source used to interrogate the porous samples at that wavelength range; for example, the interference maximum located at 525 nm provides a sensitivity of 224 nm/RIU with a noise of σ = 23.1 pm, leading to an estimated LOD of 3.09 × 10^−4^ RIU.

**Figure 5:**
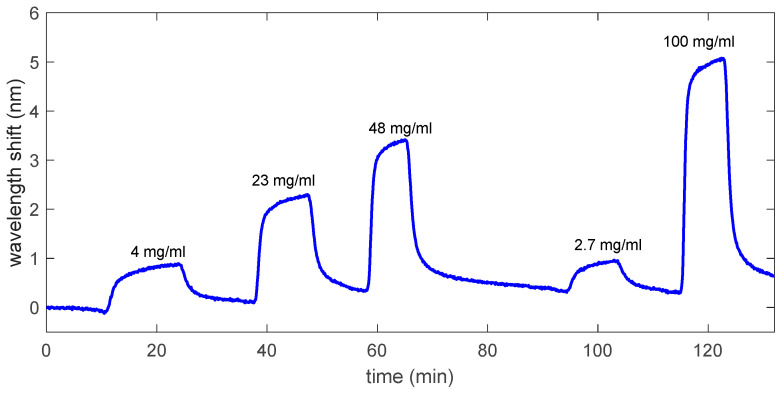
Spectral shift of the interference maximum of the reflection spectrum located at 620 nm when selected concentrations of glucose in DIW are flowed. The raw sensing curve is depicted (i.e., not averaged).

After determining the bulk sensitivity, the mesoporous TiO_2_ sample was used to detect different concentrations of glucose in DIW, ranging from 2.7 to 100 mg/mL (270 to 10,000 mg/dL). Note that the porous substrate was not modified to make it selective to glucose, hence the direct response of the TiO_2_ porous layer to that molecule was measured in order to assess the sensing potential of the fabricated layers. Figure 5 shows the evolution of the position of the interference maximum located at 620 nm when the different glucose concentrations were flowed. As it can be observed, the sensing signal for the different glucose cycles did not show a step-like behavior as in the case of ethanol, but instead showed an exponential-like shape that indicates that glucose molecules might get deposited onto the inner walls of the pores. When DIW is flowed again, an inverse exponential-like shape appears that indicates the desorption of glucose molecules from the pores. However, that desorption seems to be quite a slow process, since recovery to the initial baseline does not fully occur by the time the sensor is rinsed with DIW after each glucose cycle. A method that could improve the response time of the sensor might be to increase the flow rate during the DIW rinsing cycles so that glucose molecules are fully removed from the pores as rapidly as possible.

**Figure 6:**
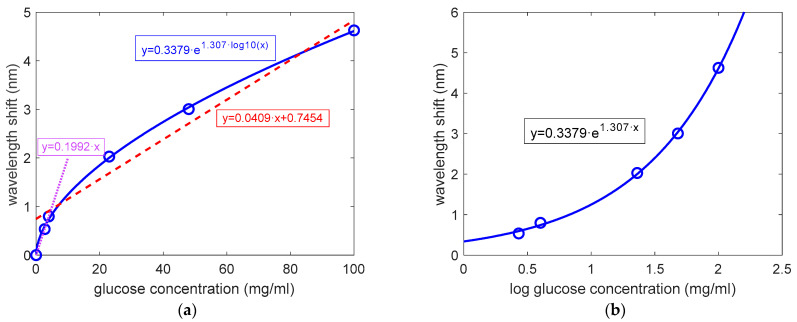
Sensitivity analysis of the glucose detection results obtained for the P123-templated mesoporous TiO_2_ tri-layer, where a time of 5 min has been considered for the stabilization of the sensing signal after the injection of each glucose cycle (experimental sensing results depicted with blue circles). (**a**) The red dashed line and the blue solid lines correspond to the fitting of the experimental sensing results to a linear function and to an exponential function, respectively. The purple dotted line corresponds to the linear fitting of the experimental sensing results for glucose concentrations below 5 mg/mL. (**b**) Sensitivity curve corresponding to the fitting of the experimental results to an exponential curve when the logarithm of the glucose concentration is considered on the x axis.

Figure 6 shows the sensitivity analysis for the glucose detection experiments carried out, where the wavelength shifted 5 min after the injection of each glucose solution was considered (volume flowed = 100 μL). This shift time was considered since the sensing signal is practically stabilized for all the glucose concentrations, although a shorter time might be used if required (the sensing signal can be considered to be close to stabilization from minute 2–3). As it can be observed in Figure 6a, the measured data poorly fit a linear function, which in addition has a significant offset when a glucose concentration of 0 mg/mL is considered (745 pm). However, measured data perfectly fit an exponential function dependent on the logarithm of the glucose concentration (R^2^ = 0.9992). Figure 6b shows this same exponential sensitivity curve when the logarithm of the concentration is considered in the x axis, as is typically done in concentration–response tests in bioassays [40,41]. This exponential sensitivity curve can be explained by the adsorption–desorption processes previously commented on. If the sensing response was only related with the bulk refractive index variation produced by each glucose concentration, a linear sensitivity curve would be obtained. However, the adsorption–desorption of glucose molecules to the pores’ walls seems to be a non-linear process that is dependent on the concentration, hence leading to the previously observed exponential-like sensitivity behavior. From considering the obtained sensitivity curve in addition to the noise level previously determined for the EtOH calibration experiment (9.8 pm), we can estimate a LOD for glucose detection as low as 0.014 mg/mL (i.e., 1.4 mg/dL).

Finally, we also considered a linear fitting of the experimental sensing data for glucose concentrations below 5 mg/mL (i.e., 500 mg/dL) (purple dotted line in Figure 6a), since a good fitting is obtained in this way. A sensitivity of 199.2 pm/(mg/mL) was determined for this concentration range when considering this linear behavior. Regarding the LOD, we can now estimate a value of 0.15 mg/mL (i.e., 15 mg/dL). This value is one order of magnitude higher than when an exponential sensitivity behavior was considered, but is still below the lowest relevant concentration in the aforementioned applications. We can also use this linear fitting to estimate the accuracy of the measurements as σ/sensitivity, leading to a value of 0.05 mg/mL (i.e., 5 mg/dL) for glucose concentrations below 5 mg/mL.

## 4. Conclusions

The potential of mesoporous TiO_2_ layers created using a bottom-up synthesis approach for optical sensing purposes has been demonstrated. This method overcomes some limitations associated with more typical top-down fabrication strategies that rely on the anodization of a bulk substrate. This method opens the door to the creation of porous optical sensing substrates that are low-cost, easy to implement, with a high reproducibility and suitable for large-scale production. The fabricated TiO_2_ layers displayed a highly interconnected mesoporous network of controlled pore size (15–20 nm) in addition to good surface homogeneity. Note also that the sample evaluated in this work for glucose sensing was obtained by sequentially depositing and aging three mesostructured layers over the same substrate, which became mesoporous after the final thermal treatment. This strategy enabled an increase in the total thickness of the layer, which provided narrower interference fringes in the reflectivity spectrum that can be more accurately tracked during the sensing experiments; however, it could also be used to create more complex optical structures, such as Bragg reflectors or microcavities, just by changing the properties of each of the deposited layers.

The fabricated mesoporous TiO_2_ layers exhibit a remarkable Fabry–Pérot optical response in the VIS-NIR range, displaying several lobes and a high reflectivity, characteristics that are suitable for sensing purposes. We have experimentally evaluated the sensing performance of these layers. First, the bulk sensing performance of the porous layers has been evaluated by flowing an EtOH solution through the sensor, obtaining a remarkable sensitivity of 260 nm/RIU for the selected interference lobe. A significantly reduced noise level was measured for these experiments (σ = 9.8 pm), leading to an estimated LOD of 1.13 × 10^−4^ RIU. This LOD value is higher than those obtained for other optical sensing approaches, such as those based in integrated photonics, but we have to take into account the relative simplicity of this type of porous sensing layer, its fabrication process and its characterization in comparison with those of other approaches. Thus, these properties make this type of sensor very practical in a wide range of applications where not such extremely low concentrations of the target analytes are required to be detected. 

Next, the porous TiO_2_ sensing layer was used for the detection of synthetic glucose samples at different concentrations ranging from 2.7 mg/mL to 100 mg/mL (270 mg/dL to 10,000 mg/dL). The measured sensing response perfectly fit to an exponential function as a function of the concentration in logarithmic scale. From this sensing response, and considering the noise level previously characterized, a LOD for glucose detection of only 0.014 mg/mL (1.4 mg/dL) was estimated. This LOD value increases up to 0.15 mg/mL (15 mg/dL) if a linear sensitivity is considered for glucose concentrations below 5 mg/mL. For any of these LOD values, the developed porous sensing layer would cover a wide range of concentrations (from 1.4 mg/dL or 15 mg/dL, to 10,000 mg/dL), which includes a range from the lowest concentration considered for glucose blood/urine level monitoring (usually found at 50 mg/dL), to the highest concentration typically considered for beverages (in the range of 7000 mg/dL).

Therefore, the combination of good sensing performance and suitability of the bottom-up fabrication process for high-volume production makes these porous layers a very promising candidate for the development of low-cost and high-performance sensors. However, there still are some practical aspects that need to be addressed and/or studied in order to ensure their applicability in the fields of interest, such as specificity and selectivity (by a proper biofunctionalization of the porous layer with specific biorecognition elements), robustness and reliability (which are expected from the properties of TiO_2_ as a constitutive material as well as from the biofunctionalization process used to avoid non-specific signals) or required sample volume and time (where an adequate balance between parameters such as flow rate, dilution factor or sensitivity must be determined), among others. These aspects will be addressed in future research.
